# Considerations for the equitable selection of thoracic surgery residents and fellows

**DOI:** 10.1016/j.xjon.2025.09.042

**Published:** 2025-10-10

**Authors:** Ahmet Kilic, Mara B. Antonoff, Kathleen S. Berfield, David T. Cooke, Thomas K. Varghese, Mark Orringer

**Affiliations:** a Department of Surgery, Johns Hopkins Hospital, Baltimore, Md; b Department of Thoracic and Cardiovascular Surgery, The University of Texas MD Anderson Cancer Center, Houston, Tex; c Department of Surgery, University of Washington, Seattle, Wash; d Division of General Thoracic Surgery, University of California, Davis Health, Sacramento, Calif; e Division of Cardiothoracic Surgery, University of Utah, Salt Lake City, Utah; f Department of Surgery, University of Michigan, Ann Arbor, Mich

**Keywords:** ethics, surgical training, the match

## Abstract

**Background:**

Equitable selection of surgical trainees is essential to maintaining the integrity and excellence that is central to cardiothoracic surgical education. Selection processes must ensure that all candidates are evaluated fairly and consistently, minimizing bias while identifying those with the greatest potential for technical competence, clinical excellence, and upholding the principles of professionalism. Despite progress, many current approaches remain overly reliant on academic metrics that do not fully capture the attributes required for success in surgical training and practice.

**Objective:**

This expert opinion article examines contemporary challenges and evolving priorities in achieving a fair and equitable selection of surgical trainees, drawing on experience in surgical education, program leadership, and performance assessment.

**Results:**

Traditional selection metrics—such as academic achievement, standardized test scores, and research productivity—remain valuable but insufficient predictors of operative aptitude, resilience, teamwork, and ethical judgment. Emerging evidence supports a more comprehensive evaluation framework that incorporates behavioral and situational assessments, structured interviews, and evaluation of professional attributes such as integrity, adaptability, and commitment to improvement. This article proposes a multidimensional selection model designed to improve predictive validity and fairness in trainee recruitment and assessment.

**Conclusions:**

The future of surgical education depends on refining selection processes to identify candidates with both technical potential and the personal characteristics essential for excellence in cardiothoracic surgery. An evidence-informed, transparent, and equitable approach to trainee selection will strengthen the specialty and ensure the continued delivery of high-quality patient care.

**Figure:**
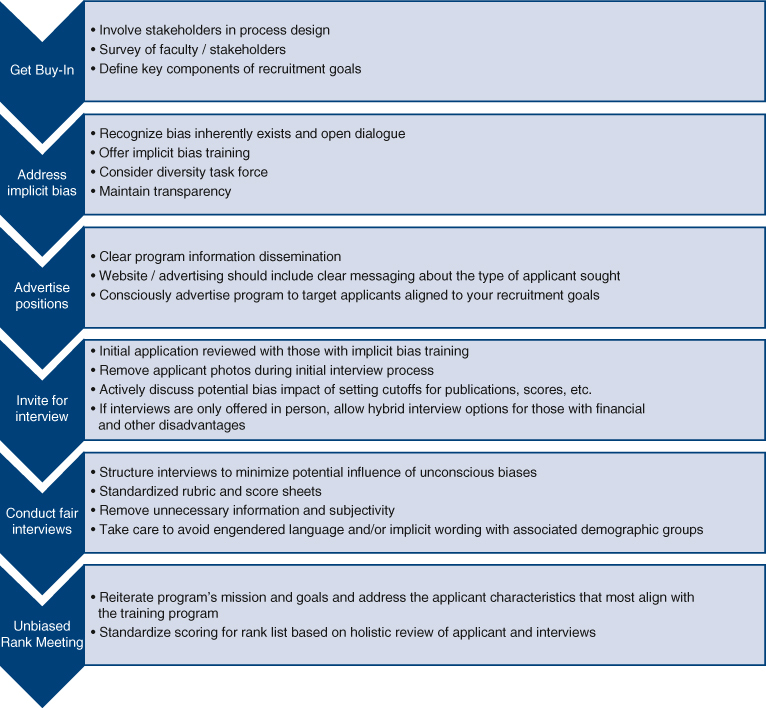
Best practices for holistic resident selection.

**Central Message**

In an ever-changing world, programs need to maintain fairness in the evaluation of applicants and the conduct of interviews, and to match the trainees who best fit their program's mission.

**Perspective**

Matching into a thoracic surgery training program, whether via the traditional or the accelerated/integrated pathway, has become more competitive in recent years. In this high-stakes, stressful environment, programs and applicants alike must strive to uphold a standard of fairness, equity, and ethics in the match process.

In 3 years, we will celebrate 100 years since Dr John Alexander established the first thoracic surgery residency in the United States at the University of Michigan in 1928. In his 1936 address before the American Association for Thoracic Surgery on “the training of a surgeon who expects to specialize in thoracic surgery,” Alexander espoused his “2-year rule” of intensive thoracic surgery training needed to produce a competent thoracic surgeon—a dictum that guided our specialty until the advent of the integrated 6-year (I-6) programs in 2007.^1^ He also enumerated his criteria for trainee selection to include only “highly desirable students,” graduates of “class A medical schools,” and those who completed general surgical training in “high-grade hospitals.” In addition, he stated it was desirable to have some training in internal medicine, sanatorium practice, anatomy, pathology, physiology, and laboratory methods; an interest and familiarity with laboratory methods (which should result in preference over other equally qualified applicants); sufficient good health to withstand the rigors of training in this specialty; temperaments and personalities likely to contribute to success in this field; and a reading knowledge of medical German (ability to read French, Italian, and Spanish were “also desirable”). If we fast forward, within 40 years, thoracic surgery residency programs in the United States were shaped and refined as the specialty saw the emergence of the Accreditation Council for Graduate Medical Education Residency Review Committee (1967), the Thoracic Surgery Directors Association (1967), the American Board of Thoracic Surgery as an independent specialty board (1971), the National Thoracic Surgery Resident Matching Program (1982), and in 2007, the advent of the I-6 and alternative pathways to American Board of Thoracic Surgery certification. However, with these developments in the structure and conduct of our residencies, what has become of Alexander's originally defined criteria for selection of trainees into the variety of paradigms for training now available—the traditional 5 + 2 year fellowship, and the I-6 and 4 + 3 year residencies? This manuscript examines the current criteria, particularly their ethical aspects, for selecting our residents and fellows.

## Traditional Selection Criteria for Trainees

**Figure 1:**
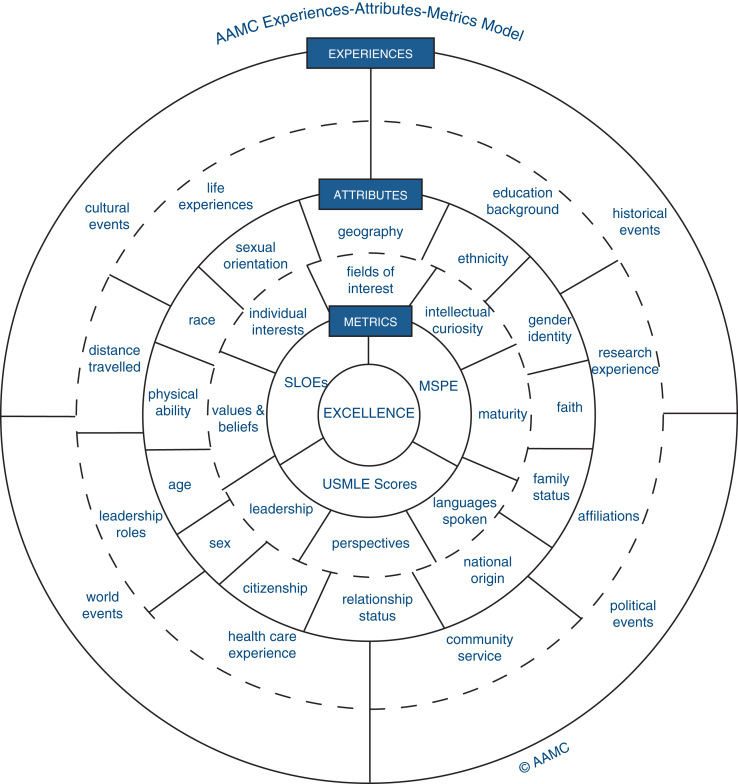
The Association of American Medical College (AAMC) Experiences–Attributes–Metrics model for the assessment of applications. (Adapted from Loden and Rosener.^2^ Reprinted with permission from AAMC July 2025). *USMLE*, United States Medical Licensing Examination.

There are 2 main thoracic surgery training pathways approved by the Accreditation Council for Graduate Medical Education: (1) the traditional pathway, which requires the completion of a general surgery (or vascular surgery) residency followed by a 2- or 3-year independent thoracic surgery training, and (2) the accelerated pathway, which is either a joint general surgery/thoracic surgery (4 + 3 years) training or an I-6 pathway. In recent years, it has become increasingly competitive to match into either of these pathways and although there are no strict “traditional criteria” used by programs during the National Thoracic Surgery Resident Matching Program, the Association of American Medical Colleges Experiences–Attributes–Metrics model is a useful blueprint to assess the merits of an application (Figure 1).^2^

### Traditional Pathway

Prospective trainees on this pathway are more “surgically mature” than those applying to the accelerated pathway. That is, the program reviews a board-eligible surgeon who has already successfully matched at least once, has had the opportunity to develop and to showcase technical skills, and to have a network of mentors within the specialty. That being said, the applications are weighted on “academic excellence” as reflected by the applicants—specifically their academic heritage—where they went to medical school and trained in residency; medical school grades; in service examination scores during residency; Unites State Medical Licensing Examination (USMLE) scores, as well as a demonstrated interest in cardiothoracic surgery. Additional accomplishments during residency that are a good indicator of academic potential in programs that favor training academic surgeons include publications and presentations during professional development years as well as degrees earned during this time period. Other accomplishments such as Alpha Omega Alpha induction, Gold Humanism Award, and teaching awards from medical students and more junior residents often resonate to the quality of the applicant. Mentors identified, networks developed, a standout personal statement, and individualized letters of recommendation are factors that can differentiate the applicant in the program's review. The objective of the program is to match with an applicant who fits into the mission and goals that are specific to that training program.

### Accelerated/Integrated Pathway

Selection of candidates for integrated thoracic surgery residency programs has become increasingly challenging for programs since 2008, when the first integrated thoracic surgery (I-6) residency programs joined the Match. In the 2025 Match, there were 36 I-6 programs and 54 available postgraduate year 1 positions. More than 225 applicants applied to an I-6 program, with the average program receiving 148 applications, which represents a 30% increase in the number of applicants over the last 5 years. The Match rate was only 24%.^3^ Historically, USMLE board scores and metrics such as Core clerkship grades have been used by programs to screen applicants on the basis of their apparent clinical knowledge and acumen. It was well recognized, however that using USMLE Step 1 scores in this capacity was flawed and has not always provided an accurate representation of applicant knowledge or clinical capability,^4^ and as a result the National Board of Medical Examiners and Federation of State Medical Boards transitioned to Pass/Fail grading for the USMLE Step 1 examination in 2022. There is also significant variability in clerkship grading across medical schools, which makes one-to-one comparisons of clerkship performance challenging for programs. These measures have made the applicant screening process less straightforward and potentially more difficult for qualified international medical graduates to obtain residency positions in the United States. Specifically, without direct comparative metrics, it is harder for an international graduate to have their application standout from their peers. As such, programs have had to invest more time in the applicant review processes and to perform more holistic reviews for all applicants.^5^^,^^6^

Programs have limited information about applicants, and unless an applicant has completed a Sub-I or is an internal candidate, they are reliant on the Electronic Residency Application Service application for applicant data. Although the Electronic Residency Application Service application is the same for all applicants, it is important to note that an institution's Graduate Medical Education (GME) department may restrict a program's ability to see certain components of the application such as the professional photo, gender, ethnicity, geographic preferences, or work authorization. As with fellowship applicant reviews, residency applicants are evaluated on the basis of medical school and previous undergraduate and graduate training, academic and clinical performance, scholarly activity and research, community service, extracurricular activities, demonstrated leadership skills, personal statement, and letters of recommendations. Although the selection criteria used by programs is largely the same, what distinguishes I-6 programs from each other is the relative emphasis each program places on individual criteria.

## Factors to Consider to Ensure the Equitable Selection and Evaluation of Thoracic Surgery Trainees

Several ethical problems exist in the realm of selection bias for trainees in cardiothoracic surgery. Qualified candidates from underrepresented groups may be systematically overlooked, representing inequitable access. Reinforcement of systemic biases remains prevalent, with selection at times determined on the basis of school prestige, reputation, or personal connections, perpetuating existing inequalities.^7^^,^^8^ Homogeneity of trainees in training programs reduces diversity of thought, background, and patient care experiences. Bias may favor less-qualified candidates over better-qualified ones, eroding fairness, and undermining meritocracy. Further, perceived, or real bias diminishes trust in the medical education system. Consequently, unfair exclusion of qualified candidates may impact patients, because a lack of diverse perspectives can negatively affect culturally competent care.^7^^,^^8^ Finally, biased selection may teach future leaders to replicate unfair practices.

### Common Language for Letters of Recommendation

Previous authors have evaluated the impact of biases on resident recruitment and have found that bias plays a role at every step of the pathway, in areas ranging from screening of applicants to the construction of letters of recommendation (LoR).^9^^,^^10^ In a multi-institutional study, investigators found that faculty members reviewing applications discriminated on the basis of race, ethnicity, and body habitus.^11^ Inconsistencies in LoR are particularly problematic. They are often the first real glimpse of the prospective candidates’ thought process and motivations for entering into the specialty. Numerous investigators have shown differences in types of letters written for men versus women applicants, as well as racial differences in content of letters.^12^^,^^13^ Within cardiothoracic surgery, a study using natural language processing to assess LoR written for fellowship applicants found substantial differences in linguistic terms used to describe male applicants compared with female applicants, despite comparable achievements and experiences.^14^ These findings are critical to consider as we conduct assessment of applicants in surgical specialties, and ongoing efforts to optimize candidate selection are strongly needed to minimize bias and to support a diverse workforce. Moreover, these data provide clear evidence for deliberate efforts to holistically review candidates in a manner immune to interference from such biases.

### Holistic Review of Applicants

**Figure 2:**
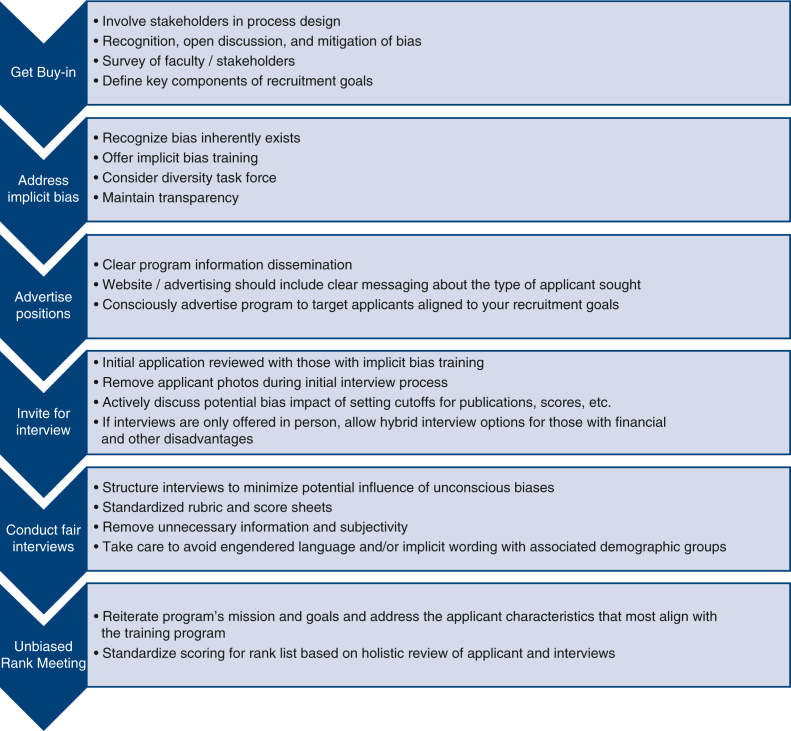
Best practices for holistic resident selection.

Holistic review considers an applicant's entire profile rather than focusing solely on metrics. Despite previous efforts by a number of individual programs to mitigate biases, there are several prevailing issues that persist: Our specialty fails to represent our patients and our communities; members of underrepresented groups continue to have disparate experiences in applying, interviewing, and securing cardiothoracic surgical training positions; and, finally, we have wide variability in practices to mitigate bias.^7^ Given these challenges, a systematic approach to best practices for holistic evaluation can be highly valuable. Such an approach has several key components (Figure 2). The overarching strategy^8^ involves the following steps: (1) get buy-in; (2) address implicit bias; (3) advertise positions widely; (4) invite for interviews equitably; (5) conduct interviews fairly; and (6) conduct rank meetings without bias.

### Considerations and Current Stressors in Applicant Selection

Current stressors in cardiothoracic surgery applicant selection include increasing scrutiny of diversity initiatives amid shifting legal and social landscapes.^15^ Although programs strive for holistic admissions and fairness, efforts to recruit women and underrepresented minorities in surgery are often met with skepticism. When residency classes are majority female or racially diverse, some critics question whether selections were merit-based, revealing a persistent double standard. Conversely, homogenous classes are rarely interrogated through the same lens.^16^ This imbalance can negatively affect belonging, placing undue pressure on both programs and applicants from historically excluded groups. Trainees often are made to feel as though their presence requires justification beyond qualifications.^17^ As we navigate legal rulings and heightened public scrutiny, programs must affirm that excellence and diversity are not mutually exclusive. Addressing both active and unconscious bias transparently is essential to upholding ethical, equitable selection practices in thoracic surgery.

### Gauging Technical Proficiency

Graduation represents a significant investment and should not be misaligned with an individual's skill set. Cardiothoracic surgery demands a high level of technical proficiency. Identifying candidates lacking the necessary dexterity or spatial reasoning early allows those candidates to pursue non-cardiothoracic surgery specialties in which they can excel; an approach that is protective of applicants, training programs, and, ultimately, patients. In other fields, upfront demonstrations of technical ability are standard: high school musicians submit audition videos for elite symphonies, and athletes provide highlight reels to collegiate recruiters. Similarly, surgical specialties are increasingly adopting video-based assessments to evaluate technical skills objectively.^18^ Implementing simulation performances, video submissions, or manual skill assessments during the cardiothoracic surgical residency/fellowship application process can serve as ethical and equitable screening tools, where the goal is to guide candidates to settings where their talents align.

## Social Media Guidelines for Applicants and Institutions: Critical Skillset or Overreach?

Social media are computer-mediated technologies that facilitate the creation and sharing of information, ideals, career interests and other forms of expression via virtual communities and networks. Across industries, 70% of companies use social media to screen job candidates, 50% rejecting applicants based on what they discover on social media.^19^ Social media has introduced new dynamics in the selection process for GME. Early studies have shown that 17% of program directors screened applicants using social networking sites, with 33% of those screened receiving lower rankings based on discovered content.^20^ Program directors and selection committees are increasingly engaging with applicants’ online personas, prompting a debate: is this a critical skillset for identifying professionalism and alignment with institutional values, or an overreach into personal domains that risk bias and inequity?

### Social Media as a Screening Tool: Rationale and Opportunities

Proponents argue that reviewing an applicant's social media presence provides insight into character, judgment, and professionalism—qualities that are essential in GME.^21^ With training programs placing a premium on team dynamics, communication, and ethical behavior, information available to the public is a legitimate extension of a candidate's portfolio. Inappropriate content, such as discriminatory remarks, breaches of patient confidentiality, or unprofessional conduct, may raise red flags. Conversely, social media may also highlight positive attributes—such as advocacy, leadership, or community engagement—that may not be fully captured in formal applications.

The modern surgeon is increasingly expected to maintain a professional digital footprint. In this context, social media literacy and digital professionalism are viewed by some as essential competencies.^22^ Educational institutions have begun incorporating digital citizenship into their curricula, emphasizing the need for students to align their public online presence with the expectations of the medical profession. For programs, assessing this dimension may reflect a broader commitment to training socially responsible, media-savvy physicians.

### Concerns About Overreach, Bias, and Fairness

Critics caution that using social media for applicant screening introduces the risk of subjective interpretation, and unconscious bias.^19^ Social media are often created for personal expression and not designed for professional purposes. Decisions based on perceived political views, lifestyle choices, or cultural expression may reflect unconscious bias rather than objective metrics of professional potential. In addition, not all applicants use social media or maintain public profiles, raising concerns about equity in evaluation and the consistency of review standards.

The potential for legal and ethical pitfalls is also significant. Without transparent policies, programs risk violating applicants’ rights or creating a chilling effect where individuals feel pressure to self-censor out of fear that personal views could jeopardize career opportunities. There is a lack of formal guidelines from governing bodies leaving programs without clear frameworks.

### Striking the Right Balance—A Call to Action

The evolving digital landscape suggests that social media will remain relevant in medical education and professional development. Programs should develop transparent, standardized policies for social media screening, ideally reviewed by institutional legal and equal opportunity offices. While there is an opportunity for organizations such as the Association of American Medical Colleges and Thoracic Surgery Directors Association to develop guidelines, equally important is educating applicants on responsible digital behavior without punishing authenticity.

In conclusion, while reviewing social media may offer a window into applicants’ professionalism and values, it must be approached with caution, structure, and fairness for all. Whether it becomes a critical skillset or remains a controversial overreach will depend on how deliberately and ethically programs integrate it into their selection process.

## Toward Clarifying and Standardizing Criteria for Thoracic Surgery Resident/Fellow Selection

John Alexander's original trainee selection criteria—only “highly desirable” students from a “class A medical school” and trained at a “high-grade hospital”—and his preference for “academically productive” applicants were largely subjective and inherently biased. The notion that preferential acceptance is linked to one's publication record encourages applicants to invest time and energy into research and publishing prior to applying. Of the 2024 matched cardiothoracic surgery (5 + 2) fellows, at the 25th, 50th, and 75th percentiles, the average numbers of research experiences were 2, 5, and 9, respectively; the average numbers of publications were 10, 17, and 32.^23^ Even among the I-6 resident applicants, with presumably fewer years to be academically productive, average research experiences were 3, 4, and 7; and publications 10, 15, and 26.5, not appreciably different. Have we overamplified Alexander's inherent bias against applicants lacking a glowing research and publication record despite little correlation between curriculum vitae length and cardiothoracic surgery career success? Are we fixated more on standardized test scores rather than other potential “markers” of talent and professional success, eg, achieving Eagle Scout rank, being a Division 1 college athlete (implicit resilience, perseverance, and time-management skills), or being a musician (practice, repetition, and perseverance)? The relative weighting of residency selection criteria cannot be standardized, as programs differ widely in their emphasis on educational, research and academic productivity priorities. We strongly recommend, however, that the evaluating faculty of each residency annually review its program's selection criteria and which warrant priority, acknowledging inherent biases based upon ethnicity, gender (of both applicants and their letter writers), demographics and prior education/training. The proportion of female cardiothoracic surgery trainees has grown from 2% in 2001, to 15% in 2007, 19% in 2011, and 26% in 2020^24^^,^^25^ but is still far from the 50% proportion of current medical students. Consensus should be reached on the use of social media profiles in screening applicants. Although cardiothoracic surgery fellowship applicants have to successfully complete a general surgery training program, a major challenge to assess the requisite technical skills of our I-6 resident applicants remains. Ongoing review and discussion of the criteria of resident/fellow selection demonstrates our specialty's commitment to minimizing bias and providing as fair and transparent a selection process as is possible.

## Conflict of Interest Statement

The authors reported no conflicts of interest.

The *Journal* policy requires editors and reviewers to disclose conflicts of interest and to decline handling or reviewing manuscripts for which they may have a conflict of interest. The editors and reviewers of this article have no conflicts of interest.
